# A Manual of the Diagnosis and Treatment of the Diseases of the Eye

**Published:** 1900-05

**Authors:** 


A MANUAL OF THE DIAGNOSIS AND TREATMENT OF THE DISEASES OF THE EYE. BY EDWARD JACKSON, A. M., M. D., Emeritus Professor of Diseases of the Eye in the Philadelphia Polyclinic; formerly Chairman of Section on Ophthalmology of the American Medical Association; Member of the American Ophthalmological Society; Fellow and Ex-President of the American Academy of Medicine. Duodecimo, with 178 illustrations and two colored plates. Pages 604. Philadelphia: W. B. Saunders, 925 Walnut Street. 1900.

With such excellent hand-books on the eye at hand as the treatise of Dr. Noyes, the text-book of Dr. de Schweinitz, the American text-book, which is distinctively American, and the American issues of Fuchs, Swanzy, Nettleship, Jessop and others so acceptably received in this country, there is, perhaps, a question as to the real need of another of the same kind. And yet who can object when one is offered by an author so well known and so well qualified as is Dr. Jackson? For many years he has contributed quite liberally to the literature of ophthalmology and his writings have taken high rank. Those on refraction are regarded as authoritative. In view of his attainments and experience and the conscientiousness with which he uses his pen, nothing but a careful and complete survey of ophthalmology would be expected in this work. And such it proves to be on examination. It is a compact duodecimo volume of 604 pages, including the index, and aims "to meet the needs of the general practitioner of medicine and the beginner in ophthalmology." First, importance is given to the recognition and management of the conditions likely to be presented early in practice, and less stress is laid upon rarer diseases and difficult operations. The subjects are arranged in order similar to that of other hand-books, and at the end of each chapter is added a selected bibliography that serves to direct the reader to more extended studies, should he desire to pursue them. To many this will be a great help. Another feature of the work, oftentimes of great convenience, is the placing of the title of the subject-matter of each page at its top.

The work is divided into twenty chapters, the last of which is very useful to the general practitioner as well as the ophthalmologist, and reviews the ocular symptoms and lesions connected with general disease. Ophthalmic operations are described in a separate chapter, and include those which one is most commonly called upon to perform. Perhaps the most complete part of the work and that in which the personality of the author is most apparent is that which deals with refraction and the diagnosis and treatment of refractive errors. The reviewer endorses, fully, the author's methods and conclusions. In emphasizing, however, the value of this part of the

"manual," there is no intention of detracting from the merit of the other chapters. The latter are well-proportioned and the subjects contained in them are considered clearly, concisely, and as fully as limited space allows. If there be any short-coming, whatever, in the work, it is in the too brief consideration of injuries to the eye; but this brevity is no less conspicuous in this than in other works of its kind. The fact is, injuries of the eyes are of prime importance to every practitioner, and our text-books should be full and explicit in dealing with their diagnosis, prognosis and treatment. If space must be curtailed, let it be in the chapters on diseases of the retina, choroid or optic nerve and not in those on injuries.

We congratulate Dr. Jackson on the production of a manual of such pronounced excellence, and we commend it to the student and the profession as a book whose statements can be trusted and whose directions can be relied upon. It is a guide of unusual value.

